# Caregivers’ hair cortisol: a possible biomarker of chronic stress is associated with obesity measures among children with disabilities

**DOI:** 10.1186/s12887-015-0322-y

**Published:** 2015-02-15

**Authors:** Xiaoli Chen, Bizu Gelaye, Juan Carlos Velez, Clarita Barbosa, Micah Pepper, Asterio Andrade, Wei Gao, Clemens Kirschbaum, Michelle A Williams

**Affiliations:** Department of Epidemiology, Harvard School of Public Health, Boston, MA USA; Centro de Rehabilitación Club de Leones Cruz del Sur, Punta Arenas, Chile; Department of Psychology, Technische Universität Dresden, Andreas-Schubert-Bau, Zellescher Weg 19, 01069 Dresden, Germany

**Keywords:** Hair cortisol, Chronic stress, Adiposity, Child, Disability, Caregiver

## Abstract

**Background:**

The stress of caring for a loved one with chronic illness has been associated with childhood obesity. Hair cortisol has been proposed as a novel biomarker of chronic psychological stress. This study aimed to evaluate the associations between caregivers’ chronic stress evaluated by hair cortisol concentrations (HCC) and obesity measures among children with disabilities such as autism.

**Methods:**

Eighty-five dyads of children with disabilities and their primary caregivers participated in the study between April and July 2013 in the Patagonia Region, Chile. Trained research staff conducted anthropometric measurements of children and caregivers. Cortisol concentrations, extracted from hair samples with methanol, were quantified using liquid chromatography tandem mass spectrometry. Pearson’s correlation coefficients and linear regression models were used to examine the associations between caregiver HCC (log-transformed) and child obesity measures with adjustment for covariates.

**Results:**

Caregiver HCC were positively and significantly correlated with child weight (child age- and sex-adjusted *r* =0.23, P = 0.036), body mass index (BMI) (*r* = 0.23, P = 0.035), circumferences of neck (*r* = 0.30, P = 0.006), waist (*r* = 0.27, P = 0.014), and hip (*r* = 0.22, P = 0.044). After adjustment for children’s age and sex, caregiver HCC were significantly related to child weight (kg) (beta = 4.47, standard error (SE) = 2.09), BMI (kg/m^2^) (beta = 1.52, SE = 0.71), neck circumference (cm) (beta = 1.20, SE = 0.43), waist circumference (cm) (beta = 3.75, SE = 1.50), and hip circumference (cm) (beta = 3.02, SE = 1.48). Caregiver HCC were also positively but not statistically significantly associated with child waist-to-hip ratio (beta = 0.01, SE = 0.01; P = 0.191) or body fat percentage (%) (beta = 2.11, SE = 1.28; P = 0.104). Further adjustment for other covariates including child disability diagnosis and caregiver age, sex, education, current smoking, perceived stress, and caregiver BMI did not change the results substantially.

**Conclusions:**

Chronic stress of caregivers, evaluated by increased cortisol concentrations in hair, was positively associated with obesity measures among children with disabilities.

## Background

Children with disabilities have higher prevalence of obesity than children without disabilities [1-7]. The 2008-2010 National Health Interview Survey showed that the prevalence of obesity among US adolescents aged 12-17 years with developmental disabilities was 20.4% as compared with 13.1% of adolescents without developmental disabilities. Among adolescents with developmental disabilities, those with autism had the highest prevalence of obesity (31.8%) [6]. Apart from unhealthy lifestyle factors (e.g., physical inactivity, poor diet), the influence of chronic stress on childhood obesity has been increasingly recognized [8-12]. Several studies have indicated a positive association between parental stress and child obesity [9,10,13]. Caregivers of children with disabilities represent a population that is known to have high levels of chronic stress [14-16]. In addition to traditional parenting responsibilities, caregivers of disabled children must also fulfill disability-related caregiving needs and therefore are prone to chronic stress [14,17]. Identifying chronic stress levels among caregivers of children with disabilities may be critical in developing effective intervention and prevention strategies to reduce childhood obesity.

Stress scales or questionnaires have been widely and typically used to capture short-term subjective stress levels, which can contribute to reporting errors [10,18]. Hair cortisol has been proposed as a novel biomarker of chronic stress that has been recently recognized as the most promising way to measure long-term cortisol synthesis and secretion for periods of several months [19-22]. Hair collection is simple and non-invasive for participants, and hair cortisol concentrations (HCC) are not influenced by moment-to-moment variations compared with other measures of cortisol from blood, saliva, or urine [20].

Body mass index (BMI) is the most commonly used measure of adiposity to describe general obesity. Because BMI does not adequately describe regional or central adiposity, other indices of body fatness such as neck circumference and waist circumference have being explored to evaluate central obesity. Neck circumference is an emerging measure of central obesity and obstructive sleep apnea [23-26]. It has been reported that long-term HCC are increased in shift workers and positively associated with BMI [27]. To date, no research has examined whether caregivers’ chronic stress evaluated by hair cortisol is associated with obesity measures among children with disabilities. This study aimed to fill the research gap by examining the associations between caregiver HCC and disabled children’s obesity measures including weight, BMI, circumferences of neck, waist, and hip, waist-to-hip ratio (WHR), and body fat percentage. We hypothesize that caregiver HCC are positively associated with child obesity measures.

## Methods

### Participants

Between April and July 2013, the Chile Pediatric and Adult Sleep and Stress Study (CPASS) was conducted with the inclusion of hair sample collection among children with disabilities and their caregivers at the Centro de Rehabilitacion Club de Leones Cruz del Sur in the Patagonia Region, Chile. Details about the study design have been described elsewhere [28]. Briefly, using a recruitment script, research staff approached primary adult caregivers when they checked in for their children’s appointment at the center. A total of 129 caregivers of children with physical and/or mental disabilities (e.g., cerebral palsy, autism) were invited to participate in the study. Ninety caregivers (including 3 caregivers with each having 2 eligible children) with 93 children (72%) agreed to participate and enrolled in the current study. Interviewer-administered questionnaires were used to collect information from primary caregivers about children’s and caregivers’ sociodemographic and lifestyle factors, as well as caregivers’ perceived stress. Children’s electronic medical records were reviewed for the confirmation of disability diagnoses and medication use. By following standardized procedures, trained research staff took anthropometric measurements twice and collected hair samples from both children and caregivers. Among enrolled participants, hair samples from 97.8% of children (two children had shaved head without hair samples collected) and from 98.9% of caregivers (one caregiver had shaved head without hair sample collected) were collected. Hair samples from 4 children and 3 caregivers were excluded because of insufficient amount of specimen. Hence, a total of 87 children and 86 caregivers (95.6% of enrolled child-caregiver dyads) completed the study protocol and were included in the present analysis.

### Anthropometric measurements

Weight (kg) and height (cm) were measured when children and caregivers were wearing light clothing without shoes. Height was measured with a telescopic height measuring instrument (Seca 225, Seca Ltd) to the nearest 0.1 cm. Weight and body fat percentage were measured with a bioelectric impedance analysis (BIA) scale (Tanita® BC 420 SMA; Tanita Europe GmbH). Weight was measured to the nearest 0.1 kg, while body fat percentage was measured to the nearest 0.1%. Circumferences (cm) of neck, waist, and hip were measured using an inelastic tape (Seca 200, Seca Ltd) to the nearest 0.1 cm, with participants in a standing position. The averages of anthropometric measurements were calculated and used in the data analysis. BMI was calculated by dividing weight (kg) by height squared (m^2^). WHR was calculated as the ratio of waist circumference divided by hip circumference.

Based on the Centers for Disease Control and Prevention (CDC) growth charts [29], children’s age- and sex-specific BMI was calculated and defined children’s overweight (85^th^ ≤ BMI < 95^th^ percentile) and obesity (BMI ≥ 95^th^ percentile). For caregivers, the World Health Organization (WHO) criteria were used to define normal weight (BMI < 25 kg/m^2^), overweight (25 ≤ BMI < 30 kg/m^2^), and obesity (BMI ≥ 30 kg/m^2^) [30].

### Hair sample collection and hair analysis

A detailed description of the methods used to measure HCC can be found elsewhere [22,31]. In brief, hair samples were cut from the posterior vertex of the scalp, as close to the scalp as possible. The most proximal 3 cm of the hair strands were used, corresponding roughly to a period of 3 months. Hair cortisol extraction procedures were similar to the methods detailed by Stalder et al. [32] with some modifications as below. Hair samples were washed in 2.5 mL isopropanol for 3 minutes and dried for at least 12 hours, after which 7.5 ± 0.5 mg of whole, non-pulverized hair was weighed out. Centrifugation was omitted since whole hair was used. Hair samples were incubated in 1.8 mL methanol for 18 hours at room temperature, and then 1.6 mL of clear supernatant was transferred into a glass vial. Subsequently, methanol was evaporated at 55°C under a steady stream of nitrogen. The residue was re-suspended using 150 μl distilled water + 20 μl of internal standard (cortisol-d_4_), 150 μl of which was used for liquid chromatography tandem mass spectrometry (LC-MS/MS) analysis. Intra-assay and inter-assay coefficients of variance were between 3.7% and 9.1% [31].

### Covariates

Child-specific factors included sex, age (years), disability diagnosis, medication use, and caregiver-reported lifestyle behaviors including diet quality, caffeinated beverage consumption, screen time, and sleep duration. According to the International Classification of Diseases (ICD-10) [33], the diagnoses of children’s disabilities were categorized as the following groups: 1) Mental and behavioral disorders, such as autism, attention deficit hyperactivity disorder, and mental retardation; 2) Diseases of the musculoskeletal system and connective tissue, skin and subcutaneous tissue, such as scoliosis; 3) Diseases of the nervous system, such as cerebral palsy; and 4) Congenital malformations, deformations and chromosomal abnormalities, such as Down syndrome. Of note, in this study, no children or caregivers had Cushing’s syndrome, a disease characterized by hypercortisolism.

Caregiver-specific factors included sex, age (years), caregiver-child relationship, marital status, education level, current smoking status, hair-related traits, perceived stress, and obesity measures. Caregivers reported their hair color, hair structure (straight or curly hair), and the use of hair treatment including coloration, bleaching, and permanent wave. Perceived stress was measured using the 14-item Perceived Stress Scale (PSS-14), which includes ratings of feeling overwhelmed, out of control, and stressed over the past month. The PSS-14 has been validated and used widely [34-36]. The PSS-14 total score ranges from 0 to 56, with higher scores indicating higher levels of perceived stress. In this study, the Cronbach alpha coefficient of the PSS-14 was 0.76, indicating that the PSS-14 had good internal consistency [37]. Caregivers in the upper quartile of the PSS-14 score ≥ 27 were considered as having higher perceived stress, while those in other quartiles (PSS-14 score < 27) were considered as having lower perceived stress.

### Statistical analysis

As this study focused on caregiver HCC and child obesity measures, we included 86 dyads of children and caregivers with complete hair data and obesity measures. One parent with extreme outlying HCC exceeding three interquartile ranges from the median and was excluded from the data analyses, leaving a final analyzed sample of 85 dyads of children and caregivers. As some children diagnosed with cerebral palsy and other disabilities were unable to stand on the BIA scale, only 70 children had measured data for body fat percentage. Note that for three families with two eligible children at each family, we chose to include one child from each family who were first enrolled in this study. We also conducted sensitivity analysis by excluding participants from these 3 families and found similar results (data not shown).

We first conducted Kolmogorov-Smirnov tests to determine the normality of caregiver HCC (exposure variable) and child obesity measures (outcome variables). As caregiver HCC were not normally distributed (Kolmogorov-Smirnov test: P < 0.05), HCC were logarithmically transformed to attain normal distribution and used in the data analyses. For descriptive purposes, we provided information on means in original units of HCC (pg/mg). Child and caregiver characteristics were presented as means and standard deviations (SDs) for continuous variables and percentages for categorical variables. Student’s t-tests were conducted to evaluate the differences in continuous variables including age and obesity measures (e.g., BMI) by sex for both children and caregivers. Chi-square tests or Fisher’s exact tests were conducted to evaluate the differences in categorical variables including disability diagnosis, medication use, sociodemographic and lifestyle factors, hair traits, and perceived stress by sex among children and caregivers. Analysis of variance or Student’s t-tests were used to assess the differences in caregivers’ log-transformed HCC across child disability diagnosis, medication use, sociodemographic and lifestyle factors, caregiver hair-related traits, perceived stress, and obesity status. Pearson’s correlation coefficients were calculated to examine the correlations between caregiver log-transformed HCC and child weight, BMI, neck circumference, hip circumference, waist circumference, WHR, and body fat percentage with and without adjustment for child age and sex.

Linear regression models were fitted to examine the associations between caregiver HCC and child obesity measures, with and without adjustment for covariates from both children and caregivers. For each outcome variable, model 1 was unadjusted; model 2 was adjusted for child age and sex; model 3 was further adjusted for child disability diagnosis and caregiver age, sex, education level, current smoking status, and perceived stress. Additional adjustment for caregiver BMI and hair-related factors including hair color and hair treatment did not change the results substantially (data not shown). By calculating Pearson’s correlation coefficients of caregiver perceived stress with caregiver HCC and child obesity measures, we conducted exploratory data analysis to determine whether caregiver perceived stress evaluated by the PSS-14 was correlated with caregiver HCC and child obesity measures.

The significance levels were set at *alpha* < 0 .05 and all reported P values are two-sided. All statistical analyses were performed using SAS® version 9.3 (SAS Institute, Inc, Cary, NC).

### Protection of study participants

This study was approved by the institutional review boards of Centro de Rehabilitacion Club de Leones Cruz del Sur in Punta Arenas, Chile and Harvard School of Public Health, USA. Because children with developmental delays such as mental retardation and motor/speech delays that would affect their ability of providing informed consent, only parents/legal guardians provided the consent for this study.

## Results

### Characteristics of study participants

Table 1 shows the descriptive characteristics of children and caregivers. The mean age of children with disabilities (boys: 42.4%) was 15.4 (SD: 2.8) years. There were no significant differences in age, disability diagnosis, medication use, lifestyle factors, weight, neck circumference, or waist circumference between boys and girls. Boys had significantly higher WHR than girls, whereas girls had higher BMI, hip circumference, and body fat percentage than boys. Based on the CDC 2000 criteria, 23.5% of children were overweight and 22.4% were obese.

**Table 1:** **Characteristics of 85 dyads of children with disabilities and their caregivers**

**Characteristics**	**Total**	**Males**	**Females**	**P value**
	**Mean (SD)/%**	**Mean (SD)/%**	**Mean (SD)/%**	
***Child characteristics***	n = 85	n = 36	n = 49	
Age (years), mean (SD)	15.4 (2.8)	15.0 (2.4)	15.6 (3.1)	0.339
Disability diagnosis^1^, %				
Mental and behavioral disorders	29.4	30.6	28.6	0.820
Diseases of musculoskeletal system & connective/skin tissue	23.5	27.8	20.4	
Diseases of the nervous system	30.6	27.8	32.6	
Congenital malformations & chromosomal abnormalities	16.5	13.9	18.4	
Medication use, %	41.2	50.0	34.7	0.157
Poor diet quality, %	30.6	38.9	24.5	0.155
Caffeinated beverage consumption, %	57.7	61.1	55.1	0.580
Screen time ≥ 2 hours/day, %	54.8	60.0	51.0	0.506
Sleep duration < 8 hours, %	32.9	38.9	28.6	0.600
Weight (kg), mean (SD)	52.9 (15.3)	52.1 (17.8)	53.4 (13.3)	0.684
BMI (kg/m^2^), mean (SD)	22.8 (5.2)	21.4 (4.4)	23.9 (5.6)	0.029
Neck circumference (cm), mean (SD)	34.3 (3.5)	35.1 (3.7)	33.7 (3.2)	0.070
Waist circumference (cm), mean (SD)	78.2 (11.0)	78.0 (11.0)	78.4 (11.2)	0.874
Hip circumference (cm), mean (SD)	91.5 (11.3)	88.4 (11.1)	93.7 (11.1)	0.031
Waist-to-hip ratio, mean (SD)	0.86 (0.07)	0.88 (0.07)	0.84 (0.07)	0.003
Body fat percentage (%), mean, SD	27.4 (9.6)	22.5 (9.4)	30.7 (8.5)	<0.001
***Caregiver characteristics***	n = 85	n = 10	n = 75	
Age (years), mean (SD)	43.1 (8.9)	41.6 (9.2)	43.3 (8.9)	0.572
Married or living with a partner, %	68.2	80.0	66.7	0.492
Education, %				
<High school	44.7	20.0	48.0	0.042
High school	28.2	20.0	29.3	
>High school	27.1	60.0	22.7	
Current smoking, %	44.7	30.0	46.7	0.501
Hair color, %				
Black	47.1	50.0	46.7	0.456
Brown	49.4	40.0	50.7	
Other	3.5	10.0	2.7	
Curly hair structure, %	36.5	30.0	37.3	0.740
Hair treatment use^2^, %	64.7	0.0	77.3	<0.001
High perceived stress^3^, %	29.4	20.0	30.7	0.716
BMI (kg/m^2^), mean (SD)	30.1 (5.2)	29.4 (1.9)	30.2 (5.5)	0.413

*Abbreviations*: *BMI* body mass index, *SD* standard deviation.

^1^Based on the International Classification of Diseases 10^th^ revision (ICD-10), the diagnosis of children’s disabilities was classified as: 1) Mental and behavioral disorders such as autism, attention deficit hyperactivity disorder, and mental retardation; 2) Diseases of the musculoskeletal system and connective tissue, skin and subcutaneous tissue such as scoliosis; 3) Diseases of the nervous system such as cerebral palsy; 4) Congenital malformations, deformations and chromosomal abnormalities such as Down syndrome.

^2^Hair treatment use included tinting, dyeing, permanent wave, and tinting pigment.

^3^High perceived stress was evaluated by the 14-item Perceived Stress Scale score ≥ 27 (upper quartile).

Student’s t-tests were conducted to evaluate the differences in continuous variables, whereas chi-square tests or Fisher’s exact tests were conducted to evaluate the differences in categorical variables by sex among children and their caregivers.

#### Distributions of caregiver HCC across child and caregiver characteristics

As shown in Table 2, there were no statistically significant differences in caregiver HCC across child sex, age, disability diagnosis, medication use, or lifestyle factors. Caregiver-child relationship, caregivers’ sociodemographic factors, smoking status, hair traits including hair color and hair treatment, perceived stress, or obesity status were not significantly related to HCC among caregivers (all P > 0.05). In total, 55.3% of caregivers were overweight while 35.3% were obese. There was no statistically significant difference in HCC among caregivers with normal weight, overweight, and obesity, although caregivers with overweight and obesity tended to have higher levels of HCC as compared to caregivers with normal weight.

**Table 2:** **Caregiver hair cortisol concentrations (HCC) across children’s and caregivers’ characteristics**

**Characteristics**	**N**	**HCC**	**P value**
		**Mean (SD)**	
***Child characteristics***			
Sex			
Boys	36	4.44 (3.06)	0.723
Girls	49	4.43 (3.38)	
Age			
11-14 years	40	4.27 (3.01)	0.270
15-17 years	31	5.17 (3.84)	
18-20 years	14	3.26 (1.81)	
Disability diagnosis^1^			
Mental and behavioral disorders	25	4.27 (3.16)	0.947
Diseases of musculoskeletal system & connective/skin tissue	20	3.85 (2.19)	
Diseases of the nervous system	26	5.15 (4.20)	
Congenital malformations & chromosomal abnormalities	14	4.22 (2.51)	
Medication use			
No	50	4.37 (3.03)	0.780
Yes	35	4.52 (3.54)	
Poor diet quality			
No	59	4.13 (2.92)	0.500
Yes	26	5.11 (3.83)	
Caffeinated beverage consumption			
No	36	4.22 (3.33)	0.313
Yes	49	4.59 (3.18)	
Screen time ≥ 2 hours/day			
No	38	4.80 (3.85)	0.396
Yes	46	4.06 (2.61)	
Sleep duration, %			
<8 hrs	28	4.58 (3.77)	0.655
8-9 hrs	33	4.67 (3.29)	
≥9 hrs	24	3.93 (2.45)	
***Caregiver characteristics***			
Caregiver-child relationship, %			
Mother	71	4.46 (3.11)	0.355
Father	8	2.93 (1.53)	
Grandmother	2	10.16 (9.39)	
Other (2 old sisters/1 old cousin/1 step-father)	4	4.05 (2.01)	
Sex			
Men	10	3.08 (1.45)	0.349
Women	75	4.61 (3.36)	
Marital status			
Married or living with a partner	58	4.50 (3.21)	0.608
Other	27	4.29 (3.33)	
Education			
<High school	38	5.48 (3.99)	0.143
High school	24	3.50 (1.62)	
>High school	23	3.69 (2.62)	
Current smoking			
No	47	3.87 (2.92)	0.109
Yes	38	5.13 (3.49)	
Hair color			
Black	40	4.55 (3.00)	0.888
Brown	42	4.40 (3.56)	
Other	3	3.46 (1.33)	
Hair structure			
Straight	54	4.15 (2.83)	0.608
Curly	31	4.92 (3.82)	
Hair treatment^2^			
No	30	3.89 (2.69)	0.432
Yes	55	4.73 (3.48)	
Perceived stress			
Low (PSS-14 score < 27)	60	4.44 (3.11)	0.749
High (PSS-14 score ≥ 27)	25	4.43 (3.56)	
Obesity status			
Normal weight (BMI < 25)	8	3.73 (2.79)	0.446
Overweight (BMI: 25-29)	47	4.23 (3.34)	
Obesity (BMI ≥ 30)	30	4.94 (3.19)	

*Abbreviations*: *BMI* body mass index, *HCC* hair cortisol concentrations, *PSS-14* the 14-item Perceived Stress Scale, *SD* standard deviation.

^1^Based on the International Classification of Diseases 10^th^ revision (ICD-10), the diagnosis of children’s disabilities was classified as: 1) Mental and behavioral disorders such as autism, attention deficit hyperactivity disorder, and mental retardation; 2) Diseases of the musculoskeletal system and connective tissue, skin and subcutaneous tissue such as scoliosis; 3) Diseases of the nervous system such as cerebral palsy; 4) Congenital malformations, deformations and chromosomal abnormalities such as Down syndrome.

^2^Hair treatment use included tinting, dyeing, permanent wave, and tinting pigment.

Student’s t-tests or analysis of variance (ANOVA) were used to assess the differences in caregiver log-transformed HCC across children’s and caregivers’ characteristics.

#### Associations between caregiver HCC and child obesity measures

**Figure 1:**
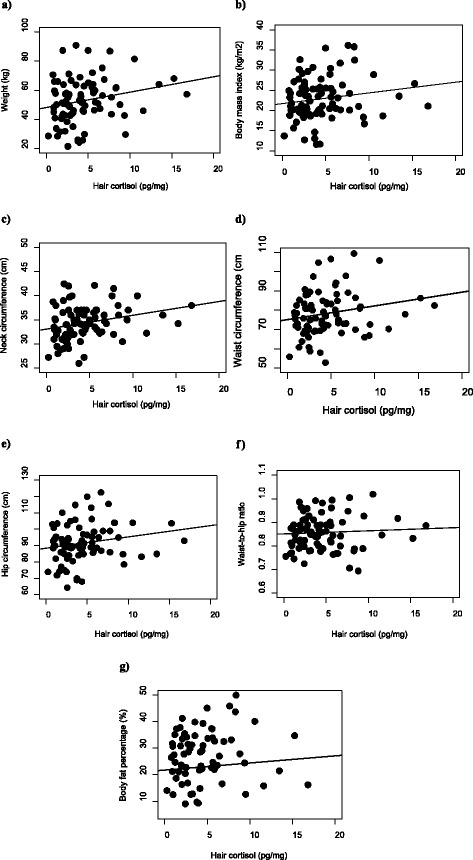
**Scatter plot of caregiver hair cortisol concentrations (HCC) by child obesity measures. a)** Pearson’s correlation coefficient for caregiver HCC (log-transformed) and child weight: *r* = 0.25, P = 0.023. **b)** Pearson’s correlation coefficient for caregiver HCC (log-transformed) and child body mass index: *r* = 0.23, P = 0.037. **c)** Pearson’s correlation coefficient for caregiver HCC (log-transformed) and child neck circumference: *r* = 0.32; P = 0.003. **d)** Pearson’s correlation coefficient for caregiver HCC (log-transformed) and child waist circumference: *r* = 0.28; P = 0.009. **e)** Pearson’s correlation coefficient for caregiver HCC (log-transformed) and child hip circumference: *r* = 0.23; P = 0.037. **f)** Pearson’s correlation coefficient for caregiver HCC (log-transformed) and child waist-to-hip ratio: *r* = 0.14; P = 0.193. **g)** Pearson’s correlation coefficient for caregiver HCC (log-transformed) and child body fat percentage: *r* = 0.16; P = 0.188.

Figure 1 shows the scatter plots of caregivers’ HCC by children’s obesity measures. There were significant correlations between caregiver HCC and child weight (Figure 1a) (*r* = 0.25, P = 0.023), BMI (Figure 1b) (*r* = 0.23, P = 0.037), neck circumference (Figure 1c) (*r* = 0.32, P = 0.003), waist circumference (Figure 1d) (*r* = 0.28, P = 0.009), and hip circumference (Figure 1e) (*r* = 0.23, P = 0.037). Caregiver HCC were positively but not significantly related to child WHR (Figure 1f) (*r* = 0.14, P = 0.193) or body fat percentage (Figure 1g) (*r* = 0.16, P = 0.188).

As shown in Table 3, after adjustment for child age and sex, caregiver HCC were positively and significantly related to child weight (*r* = 0.23, P = 0.036), BMI (*r* = 0.23, P = 0.035), circumferences of neck (*r* = 0.30, P = 0.006), waist (*r* = 0.27, P = 0.014), and hip (*r* = 0.22, P = 0.044). However, caregiver HCC were positively but not statistically significantly associated with WHR (*r* = 0.15, P = 0.191) or body fat percentage (*r* = 0.20, P = 0.104).

**Table 3:** **Pearson’s correlation coefficients between caregiver hair cortisol concentrations and child obesity measures**

**Child obesity measures**	**Unadjusted**		**Child age- and sex-adjusted**	
	***r***	**P value**	***r***	**P value**
Weight (kg)	0.25	0.023	0.23	0.036
Body mass index (kg/m^2^)	0.23	0.037	0.23	0.035
Neck circumference (cm)	0.32	0.003	0.30	0.006
Waist circumference (cm)	0.28	0.009	0.27	0.014
Hip circumference (cm)	0.23	0.037	0.22	0.044
Waist-to-hip ratio	0.14	0.193	0.15	0.191
Body fat percentage (%)^1^	0.16	0.188	0.20	0.104

Pearson’s correlation coefficients were calculated based on the log-transformed hair cortisol concentrations.

^1^Only 70 children had measured data for body fat percentage.

Linear regression models show that caregiver HCC were positively associated with child obesity measures including weight, BMI, and circumferences of neck, waist, and hip (Table 4). Caregivers’ HCC were also positively but not significantly related to WHR or body fat percentage. After adjustment for children’s age and sex, caregiver HCC were positively and significantly related to child weight (kg) (beta = 4.47; standard error (SE) = 2.09), BMI (kg/m^2^) (beta = 1.52; SE = 0.71), neck circumference (cm) (beta = 1.20; SE = 0.43), waist circumference (cm) (beta = 3.75; SE = 1.50), and hip circumference (cm) (beta = 3.02, SE = 1.48). These associations persisted after further adjustment for child disability diagnosis and caregiver age, sex, education, current smoking, and perceived stress.

**Table 4:** **Linear regression models for the associations between caregiver hair cortisol concentrations and child obesity measures**

**Child obesity measures**	**Beta (standard error)**	**P value**	**R-square**	**Adjusted R-square**
**Unadjusted models**				
Weight (kg)	4.86 (2.10)	0.023	0.06	0.05
Body mass index (kg/m^2^)	1.53 (0.72)	0.037	0.05	0.04
Neck circumference (cm)	1.43 (0.47)	0.003	0.10	0.09
Waist circumference (cm)	4.03 (1.50)	0.009	0.08	0.07
Hip circumference (cm)	3.32 (1.57)	0.037	0.05	0.04
Waist-to-hip ratio	0.01 (0.01)	0.193	0.02	0.01
Body fat percentage (%)	1.87 (1.40)	0.188	0.03	0.01
**Child age and sex adjusted models**				
Weight (kg)	4.47 (2.09)	0.036	0.10	0.07
Body mass index (kg/m^2^)	1.52 (0.71)	0.035	0.12	0.09
Neck circumference (cm)	1.20 (0.43)	0.006	0.29	0.26
Waist circumference (cm)	3.75 (1.50)	0.014	0.12	0.09
Hip circumference (cm)	3.02 (1.48)	0.044	0.19	0.16
Waist-to-hip ratio	0.01 (0.01)	0.191	0.13	0.09
Body fat percentage (%)	2.11 (1.28)	0.104	0.22	0.19
**Child age, sex, and disability diagnosis and caregiver age, sex, education, smoking, and perceived stress adjusted models**				
Weight (kg)	4.73 (1.96)	0.018	0.37	0.27
Body mass index (kg/m^2^)	1.56 (0.72)	0.033	0.29	0.17
Neck circumference (cm)	0.98 (0.44)	0.029	0.40	0.30
Waist circumference (cm)	3.74 (1.60)	0.023	0.19	0.06
Hip circumference (cm)	2.98 (1.54)	0.057	0.30	0.18
Waist-to-hip ratio	0.01 (0.01)	0.198	0.22	0.09
Body fat percentage (%)	1.89 (1.37)	0.175	0.34	0.20

Caregiver hair cortisol concentrations were log-transformed and considered as an exposure variable, while child obesity measures served as outcome variables in the models.

Caregiver perceived stress as evaluated by the PSS was not significantly correlated with caregiver HCC. Pearson’s correlation coefficient for caregiver PSS score and HCC (log-transformed) was 0.03 (P = 0.777). Caregivers’ perceived stress was not significantly correlated with child obesity measures. Child age- and sex- adjusted Pearson’s correlation coefficients between caregiver PSS score and child obesity measures ranged between 0.02 and 0.14 (all P > 0.05, data not shown).

In addition, we conducted sensitivity analysis by excluding two primary caregivers who were grandmothers, and found similar results (data not shown in tables). For example, after excluding two grandmothers and with adjustment for child age and sex, caregiver HCC were still positively and significantly associated with children’s weight (*r* = 0.23, P = 0.036), BMI (*r* = 0.25, P = 0.022), neck circumference (*r* = 0.28, P = 0.011), waist circumference (*r* = 0.27, P = 0.015), and hip circumference (*r* = 0.23, P = 0.035).

## Discussion

Hair cortisol has been increasingly recognized as a promising biomarker of chronic psychological stress that can help to understand the effects of chronic stress on health outcomes such as obesity. In this cross-sectional study, we examined caregiver chronic stress evaluated by HCC and its associations with body weight, BMI, neck circumference, and other obesity measures among children with disabilities that have not been previously characterized in these two vulnerable populations. We found that caregiver HCC were positively and significantly associated with child weight, BMI, and circumferences of neck, waist, and hip. These associations were independent of caregiver BMI and other covariates from both children and caregivers. To our knowledge, this is the first study to examine caregivers’ chronic stress evaluated by HCC in relation to general and central adiposity measures among children with disabilities. Chronic stress in mothers or other caregivers may be an important risk factor for the obesity epidemic among children with disabilities.

Identifying parental risk factors may inform intervention and prevention strategies for childhood obesity. Several studies have examined the association between parental stress and child obesity measures with inconsistent findings [9,10,13,38-43]. For example, in a cross-sectional study with parent-reported weight and height for children and one general stress question for parents, Parks et al. found that the number of parent stressors was significantly associated with child obesity (adjusted odds ratio (OR) = 1.12; 95% confidence interval (CI) = 1.03–1.23), while parent-perceived stress was related to child fast-food consumption (adjusted OR = 1.06; 95% CI = 1.02–1.10) but not significantly associated with child obesity (adjusted OR = 1.04; 95% CI = 0.99-1.09) after adjustment for covariates including child age and sex and parent education and BMI [10]. Another study conducted in a pediatric obesity treatment-seeking sample showed that self-reported parenting stress evaluated by the Parenting Stress Index for parents of children ages 3 months to 10 years and Stress Index for Parents of Adolescents aged 11-19 years did not significantly predict youth BMI [42]. A prospective cohort study of pre-adolescent children over 4 years of follow-up in southern California demonstrated a small effect of reported parental stress, as measured using the 4-item version of the PSS among parents on BMI (a two-standard deviation increase in parental stress at study entry was related to an increase in predicted BMI attained by age 10 of 0.29 kg/m^2^) [13]. One major limitation of previous stress studies was exclusively based on subjectively reported stress. HCC have been proposed as a promising biomarker of chronic psychological stress [41]. Stalder et al. recently reported elevated hair cortisol levels among 20 chronically stressed dementia caregivers compared with 20 non-caregiver controls [44]. However, no research has attempted to apply hair cortisol among caregivers of children with disabilities. Our study using a possible biomarker of chronic stress for caregivers extends previous research showing that caregiver stress is positively associated with child obesity measures. Our findings are also in agreement with results from Stalder et al. and other researchers reporting positive associations between HCC and at least one obesity measure such as BMI and waist circumference in adult populations [27,32,45,46].

There are inconsistent findings from published studies regarding associations between cortisol parameters and BMI or central obesity [47,48]. Abraham et al. reported that salivary cortisol concentrations were related to increased BMI and waist circumference in men only [47]. Rosmalen et al. reported a weak but positive association between salivary cortisol secretion and BMI in girls only [48]. These inconsistencies may be partly due to the fact that previous studies are heterogeneous in terms of study designs, study populations, participants’ sociodemographic factors, obesity measures (e.g., BMI, waist circumference), assay measurements, and cortisol parameters from blood, saliva, urine, or hair.

As BMI does not discriminate between muscle and fat mass, neck circumference and waist circumference are commonly used to measure central adiposity. Most stress and obesity studies have focused on one or two obesity measures such as BMI or waist circumference [49]. Few studies have applied multiple anthropometric indices to evaluate the association between stress and adiposity. Although neck circumference has been widely used to evaluate central obesity and sleep apnea risk factors, to our knowledge, no research has incorporated the obesity measure in the stress studies. Our study provides support for the fact that neck circumference is a simple and inexpensive measure of central obesity among children [26]. Future large studies are needed to investigate potential effect modification of child sex for the non-significant associations between caregiver HCC and child WHR and body fat percentage.

Stress is associated with an elevated secretion of hormones from the hypothalamus-pituitary-adrenal (HPA) axis. The stress response may involve metabolic changes that could directly increase adiposity [50]. There is some evidence that chronic stress may affect food choice by increasing preferences for high fat, energy-dense foods [49,51]. Stress has also been shown to reduce participation in leisure time physical activity [10], which can potentially lead to positive energy balance [49]. However, it is unknown whether relatively high cortisol concentrations in adult caregivers are associated with obesity among children with disabilities. The family stress model illustrates that parental stress can shape parenting behaviors [52], which in turn can influence child health outcome such as obesity. The relationships of parenting stress to childhood obesity among children with disabilities have received much less attention and merit further exploration. Parenting stress may contribute to the development and maintenance of child obesity due to the challenges caregivers of children with disabilities have with their children’s lifestyle behaviors. Caregivers who experience chronic stress may spend less time with their children, use less effective parenting approaches, have more challenges in shopping for fruits and vegetables, are less likely to cook healthy meals, and are more likely to purchase convenience foods which are typically high in sugar and fat (energy-dense foods) for their children [10,18]. Stressed parents may be less likely to be physically active and encourage their children to engage in leisure time exercise [10]. Parental chronic stress may be related to short sleep and sleep disturbances, which may be also associated with obesity among both caregivers and their children with disabilities. Further research is warranted to explore these associations.

Our study results showing caregiver chronic stress evaluated by HCC is associated with child obesity measures have public health and clinical implications. Efforts to reduce maternal and other caregiver stress may be particularly important for child health in families of children with disabilities. Health professionals involved in clinical care and research on childhood obesity should consider the effects of caregiver stress in order to develop effective intervention and prevention strategies and to increase stress management skills among caregivers of children with disabilities. Some policies and programs may help to reduce childhood obesity by providing support and counseling to mothers and other caregivers with high levels of chronic stress in the families of children with disabilities.

This study was the first attempt to utilize a possible, novel biological marker of chronic stress among primary caregivers of children with disabilities. The strengths of the study include chronic stress evaluated by HCC, multiple measures of adiposity, and rigorous analytic approaches. Our study also has limitations. First, as this was a cross-sectional study, our results do not provide evidence for a causal association. Further research is warranted to investigate whether increased HCC among caregivers lead to child obesity or whether obesity of children with disabilities could increase stress levels among caregivers and thus contributes to cortisol excess for caregivers. There may be also a bidirectional association between elevated cortisol concentrations and obesity. Second, we had no information on puberty for children, which might affect cortisol patterns and obesity measures. Third, our findings were based on a convenience sample with a relatively small number of children with disabilities and their caregivers, which may have limited the statistical power to detect the significant association for children’s body fat percentage. Fourth, our study focused on children with disabilities and their caregivers in Chile, and our findings may not be generalizable to other populations. The vast majority of primary caregivers (90.6%) were overweight or obese (BMI ≥25 kg/m^2^) (overweight: 55.3%; obesity: 35.3%). Furthermore, unmeasured or residual confounding is also possible, although a number of covariates were considered in our study. More research (e.g., longitudinal study design) is needed regarding the influence of caregiver chronic stress on child body composition, as well as on the interaction with lifestyle factors such as sleep traits among children with disabilities and their caregivers.

Previous findings on the correlations of HCC with self-reported perceived stress scores have been mixed with some studies reporting correlation coefficients of less than 0.10, while others showing correlation coefficients of 0.20 or more [46]. In this study, we found that caregiver HCC was not significantly correlated with caregiver PSS score (correlation coefficient: 0.03), consistent with some of previous findings [46]. In addition, we found that caregiver perceived stress was not significantly correlated with child obesity measures (correlation coefficients ranged from 0.02 to 0.14). These exploratory data analysis results are likely attributable to the fact that the PSS indicates the perceived stress levels over the past one month (a short time frame), whereas hair cortisol has been proposed as a promising biomarker of chronic psychological stress representing objectively measured stress for several months (a longer time frame than that assessed by the PSS).

## Conclusions

Our study provides the first evidence on the positive associations between caregivers’ chronic stress evaluated by cortisol concentrations in hair and obesity measures among children with disabilities. Longitudinal studies are warranted to examine the causal relationship. Future research on childhood obesity should explore the potential benefits of addressing chronic stress among caregivers and whether long-term cortisol concentrations provide a novel target of obesity prevention and treatment. It is also important to study how to improve stress resilience and stress management skills among mothers and other caregivers of children with disabilities.

## Abbreviations

BMI: Body mass index
CDC: The Centers for Disease Control and Prevention
CI: Confidence interval
CPASS: The Chile Pediatric and Adult Sleep and Stress Study
ELISA: Enzyme-linked immunosorbent assay
HCC: Hair cortisol concentrations
HPA: Hypothalamus-pituitary-adrenal
ICD: The International Classification of Diseases
LC-MS/MS: Liquid chromatography tandem mass spectrometry
OR: Odds ratio
PSS-14: The 14-item perceived stress scale
SD: Standard deviations
WHO: The World Health Organization
WHR: Waist-to-hip ratio
